# Prevalence of, Clinical Presentation of, and Factors Associated With Advanced Pelvic Organ Prolapse Among Young Women (18-49 Years) Undergoing Surgery in Urogynecologic Camps in Uganda

**DOI:** 10.7759/cureus.111302

**Published:** 2026-06-22

**Authors:** Kalyebara Paul Kato, Musa Kayondo, Onesmus Byamukama, Moses Cherop, Nikolai Hodel, Rogers Kajabwangu, Brenda Ainomugisha, Joseph Ngonzi, Verena Geissbuehler

**Affiliations:** 1 Obstetrics and Gynaecology, Mbarara University of Science and Technology, Mbarara, UGA; 2 Obstetrics and Gynecology, Mbarara University of Science and Technology, Mbarara, UGA; 3 Obstetrics and Gynecology, Soroti University, Soroti, UGA; 4 University of Basel, Swiss Tropical and Public Health Institute, Basel, CHE; 5 Obstetrics and Gynaecology, University of Basel, Basel, CHE

**Keywords:** pelvic organ prolapse, rural uganda, surgery, urogynaecology, young women

## Abstract

Background: In low-resource settings where pelvic organ prolapse (POP) surgery is often accessed through outreach surgical camps, it is crucial to have evidence of the magnitude, clinical presentation, and profile of young women with advanced POP for community health education, screening, resource mobilization, and surgical planning. The objective of the study was to describe the clinical presentation and determine the prevalence of and factors associated with advanced stage POP among young women undergoing POP surgery in urogynecologic camps in Uganda.

Methods: A retrospective records review for women aged 18 to 49 years that had undergone POP surgery in 30 urogynecologic care camps at eight centers in Uganda from January 2022 to December 2025 was conducted. The primary outcome was advanced POP defined as stage III or IV genital prolapse at maximum Valsalva strain during a speculum pelvic examination as per the POP quantification (POP-Q) system. Non-advanced POP referred to stage II POP in this study population of young women operated for POP during the study period. Predictor variables were socio-demographic, obstetric/gynecologic, and medical factors. Baseline characteristics were summarized in frequency tables. Prevalence of advanced POP was computed as a percentage with 95% confidence interval (CI). Bivariable and multivariable modified Poisson regression analysis with robust standard errors was done to determine factors associated with advanced POP. Prevalence ratios (PRs) were reported and p values less than 0.05 were considered significant.

Results: Out of 547 women operated for POP, nearly half 49% (n = 268) were young women aged 18 to 49 years and they were all enrolled into the study. They had a mean age of 38.9 years (SD: 6.9), mean parity of 5.6 (SD 2.6), presented late with a median duration of symptoms of 36 months (interquartile range (IQR) 24-84). Majority of the young women presented with the classic symptom of sensation of a mass or bulge in the vagina (84%, n = 225), concurrent pelvic floor disorders were: obstetric anal sphincter injuries (7.8%, n = 21) and stress urinary incontinence (3.0%, n = 8). The prevalence of advanced POP was 84% (224/268) (CI: 78.63 - 87.57). History of prior POP surgery increased the likelihood for advanced POP (adjusted PR (aPR) 1.19, CI: 1.08-1.31, p < 0.001), while having completed primary education (aPR 0.90 CI: 0.82 - 0.98, p = 0.015) and being married (aPR 0.91 CI: 0.85-0.98, p = 0.009) were protective against advanced POP.

Conclusions: The prevalence of advanced POP among young women undergoing POP surgery in Uganda is high. Prior POP surgery increases the likelihood for advanced POP while education and being married are protective. We recommend intensifying preventive strategies against POP in young women such as scaling up family planning uptake, skilled obstetric care, and postpartum pelvic floor muscle rehabilitation. Efforts should be undertaken to increase access and use of vaginal pessaries as first line therapy for management of POP in young women in Uganda to lower the risk of recurrence of advanced POP.

## Introduction

Pelvic organ prolapse (POP) signifies defective pelvic organ structural support in predisposed individuals leading to descent of the affected organ(s) into the vaginal cavity or through the pelvic floor [1]. POP quantification (POP-Q) system is used internationally to grade POP into four stages (I to IV) of increasing severity of descent of the prolapsed organ into or out of the vagina in relation to the hymenal ring on Valsalva maneuver during pelvic examination [1]. A degree of genital prolapse is universal to parous women and only symptomatic or advanced (stages III and IV) POP are considered pathological necessitating medical intervention [1,2].

Studies from developed countries show that the prevalence of POP rises as the population ages: from 6% for women under 30 years to 50% for women aged 80 years and above in the United States [1,3]. Similarly, the proportion of women with symptomatic POP and the rate of seeking surgical care for POP increase with age, and are highest after 65 years of age [1,4]. By the age of 80, the lifetime risk of an Australian woman for at least one POP surgery is estimated to be 19% [1]. In the developing countries, an analysis of the global burden of disease studies (1990-2019) noted higher incident cases of POP in younger women compared to developed countries [4]. A study from south-west Ethiopia found relatable results among 92 women operated upon at a university medical center between 2016 and 2018 for stage III and IV POP where more than half (53.3%) of the studied women were young (20-49 years) [5]. These reports of dissimilar burden of POP across various settings and between age groups probably reflect differences in socio-economic and reproductive risk factors for POP which therefore should be a subject of periodic context-specific research in tandem with the ever-changing lifestyles, population structure and advancements in health care.

Individual studies from African settings have reported the prevalence of POP to be: 15% for POP necessitating surgery among rural women in a community survey in Gambia [6], 19.8% among gynecology clinic attendees at two referral hospitals in Ethiopia [7], and 31.2% among women treated for pelvic floor disorders at a referral hospital in Uganda between 2014 and 2018 [8]. Studies that focus on the more clinically relevant advanced stage (III and IV) POP among young women are still limited.

POP is a stigmatizing condition and previous studies have shown that women with anatomic POP confirmed on gynecologic examination may not volunteer any of the traditionally recognized symptoms of perception of a bulge in the vagina or protrusion of the uterus through the introitus [6,9,10]. Women that have been socialized to normalize genital alterations following vaginal delivery do not link their symptoms to POP and studies show that they present with an array of complaints [6]. Additionally, POP frequently occurs concurrently with other pelvic floor disorders that may alter its clinical presentation [9]. Establishing the clinical profile of young women with POP is important in focusing community health education, mobilization, and screening, particularly in the setting where POP surgery is mainly accessed through community outreach surgical camps.

Previous studies have identified genetic predisposition with factors such as Caucasian race and family history of genital prolapse setting the intrinsic vulnerability to POP; inciting factors that initiate weakening of the pelvic organ supports such as pelvic surgery, pregnancy or childbirth; promoting factors that act through increasing intra-abdominal pressure; decompensating factors such as aging and menopause; and intervening factors such as pelvic floor exercises and body weight control [1,4,11-13]. The role of individual socio-demographic, reproductive and medical factors in this causal web of POP is not consistently demonstrated, for instance some studies emphasize the first vaginal birth as the important predictor for POP instead of multiparity while others have not found an association between parity and POP [1,11].

Therefore, the current study aimed at describing the clinical presentation and determining the prevalence of and factors associated with stage III and IV POP among young women (18-49 years) undergoing POP surgery in urogynecologic camps in Uganda.

## Materials and methods

Study design, study population, and setting

This was a retrospective review of records of young women aged 18-49 years that underwent surgery for POP in urogynecology camps in Uganda between January 2022 and December 2025. The study was conducted in eight urogynecological care centers in Uganda, namely: Mbarara Regional Referral Hospital, Jinja Regional Referral Hospital, Lira Regional Referral Hospital, Hoima Regional Referral Hospital, Bwindi Community Hospital, Ishaka Adventist Hospital, Nakaseke General Hospital, and Buyinja Health Centre. The urogynecological care team from Mbarara University of Science and Technology (MUST), with support from the local hospital teams and visiting surgeons, conducted a total of 30 urogynecology surgical camps during the study period. The urogynecologic care program transitioned from a paper-based records system that had been in place from 2009 - 2021 and set up an electronic database in January 2022 which is prospectively updated and it captures research data for quality improvement and generating scientific evidence.

Sample size

To estimate the minimum sample size required to determine the prevalence of advanced stage POP among young women (18-49 years), we used the single population proportion formula

$n = Z^2p(1-p) / d^2$

where n stands for the required sample size, z represents the standard normal deviate for 95% confidence interval (CI) and equals 1.96, d represents the desired precision of 5%, p represents the expected prevalence, and for this study, a proportion of 89.3% (proportion of anatomic uterine prolapse among women of reproductive age with prolapse symptoms) was used from an earlier Ethiopian study [14]. The substitution into the formula and sample size calculation were done in STATA software version 17 (StataCorp LLC, USA) with the command: “display (1.96^2^ * 0.893 * (1-0.893)) / (0.05^2^)” and gave a minimum sample size of 147 women. However, this study being a retrospective review, all available data of 268 women were used for the analysis. 

Inclusion and exclusion criteria

We included data of all women who: (a) were aged 18 to 49 years, (b) had provided informed consent allowing their anonymized clinical data from the urogynecology electronic database to be used for research, (c) had POP stage classified at speculum pelvic examination using POP-Q, and (d) had undergone POP surgery during the study period (January 2022-December 2025). We planned to excluded data of women where the POP-Q stage was not recorded but found none.

Study variables

The primary outcome variable was advanced POP defined as stage III or stage IV POP as determined at speculum pelvic examination on maximum straining. The stage of POP considered was from the vaginal compartment that had prolapsed the most. Our standard operating procedure for POP staging included: asking the patient to empty the bowel and bladder voluntarily before pelvic examination, positioning the patient in lithotomy, measurement of dimensions of support, followed by measurement of surface prolapse points at maximum Valsalva maneuver using a calibrated surgical ruler and Sim's speculum for retraction, and finally, documentation of the measurements in cm. POP staging was performed by senior residents of obstetrics and gynecology, urogynecology fellows in training, and urogynecologists that attended to the individual patients.

**Figure 1:**
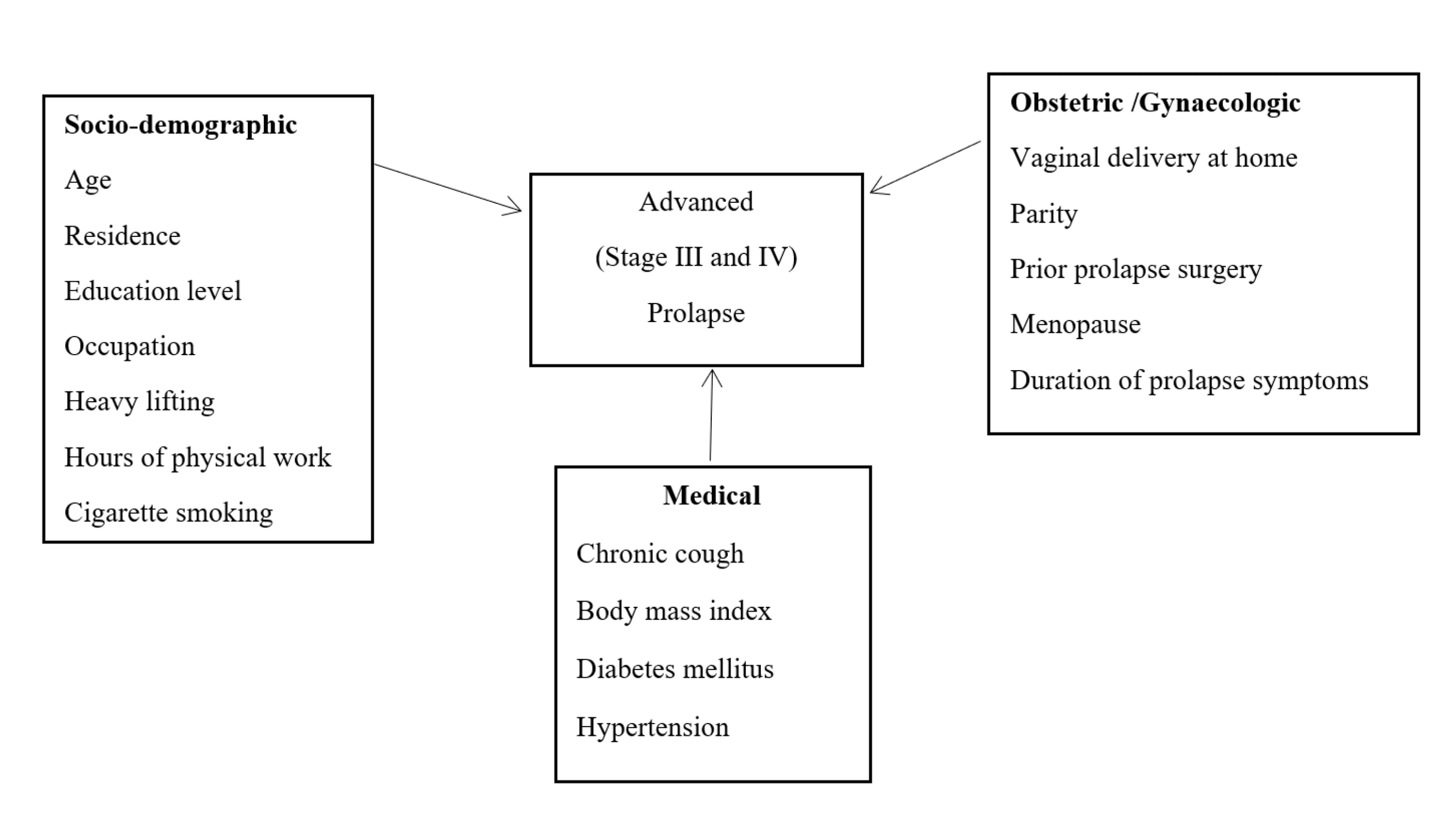
Conceptual framework for factors associated with advanced POP in young women The conceptual framework depicts that development of advanced POP is multifactorial and the contributing factors are socio-demographic, medical, and obstetric/gynecologic factors. Figure created on Microsoft Word (Microsoft Corp, USA) on Windows 11. POP: Pelvic organ prolapse

Predictor variables considered were arrived at after an extensive review of literature and a review of available data in the urogynecology database of MUST. These were compiled into a conceptual framework of socio-demographic, medical and obstetric/gynecologic factors (Figure 1).

The highest level of education attained as self-reported by the study participants was considered. Heavy lifting was a self-report of doing work that included lifting loads such as farm produce, house chores like fetching water, carrying firewood and digging, operating manual machinery, or vending merchandise in markets. Chronic cough was a report of cough persisting for a period of three months or more in the past year. Menopause was determined base on clinical history of amenorrhea lasting 12 consecutive months in the absence of an obvious pathology, history of hysterectomy or extended hormonal contraceptive use. Hypertension was based on systolic blood pressure of ≥140 mmHg or more, or diastolic blood pressure of ≥90 mmHg or more. Diabetes mellitus was based on clinical history of diagnosis with elevated blood sugar and verified from treatment records.

Data collection and management

Data was extracted from the urogynecology database for surgical camps which uses Research Electronic Data Capture (REDCap Software version 5.29.1) tools hosted at MUST. The data was exported into a Microsoft Excel spreadsheet (Microsoft Excel LTSC, version 2108) for visualization and cleaning, coded and exported to Stata version 17.0 (StataCorp LLC, USA) for analysis.

Statistical analysis

Baseline characteristics were summarized using frequency tables. Percentages were presented for categorical variables, mean and SD for normally distributed continuous variables, and median and interquartile range (IQR) for continuous non-normal distributed data.

Prevalence of advanced stage POP was computed as a percentage with 95% CI and presented as a pie-chart.

Bivariable and multivariable modified Poisson analysis with robust standard errors was done to determine factors associated with advanced POP. Exposure variables with a p-value < 0.2 at bivariable analysis were considered for multivariable analysis. Modified Poisson regression analysis was chosen due to the high prevalence of advanced stage POP found in this study. Estimates are more robust using this analysis compared to logistic regression analysis where estimates become inflated as the prevalence exceeds 10%. The level of significance at analysis was set at < 0.05.

## Results

**Figure 2:**
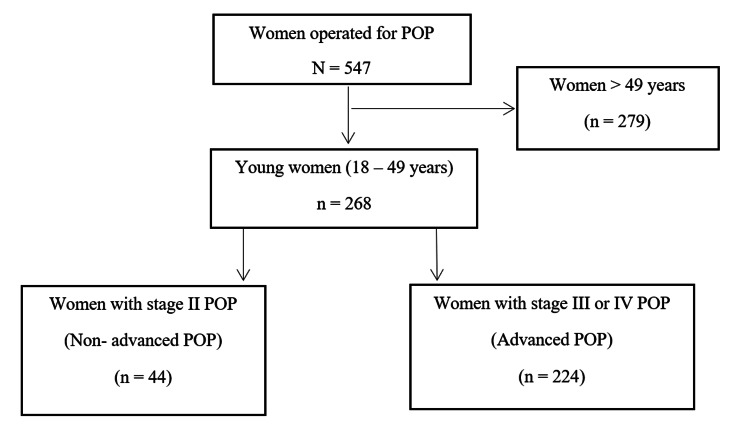
Flow chart for data retrieval and inclusion Data of 279 women was screened out as they were older than 49 years. The figure was created in Microsoft Word on Windows 11 POP: Pelvic organ prolapse

Of 547 women operated for POP, 268 (49%) were young women 18 to 49 years of age and were included in this analysis for factors associated advanced POP (Figure 2).

Out of 268 young women, the majority were aged 40-49 years (n = 142, 53%), were from rural areas (n = 250, 93.3%), were married (n = 198, 73.9%), and were peasant farmers (n = 209, 78%) as shown in Table 1.

**Table 1:** Baseline socio-demographic characteristics (n = 268)

Variable	Frequency (n = 268)	Percentage (%)
Age (years), mean (SD)	38.9 (6.9)	
Age categories		
< 40	126	47
≥ 40	142	53
Region of Uganda		
Central	20	7.5
Eastern	17	6.3
Northern	129	48.1
Western	97	36.2
East Africa	5	1.9
Residence		
Urban	18	6.7
Rural	250	93.3
Highest level of education		
Post primary	56	20.9
Primary	116	61.9
No formal education	46	17.2
Marital status		
Married	198	73.9
Widowed	11	4.1
Single, divorced, or separated	59	22
Occupation		
Peasant farmer	209	78
Other (small businesses, salaried jobs, etc.)	59	22

For the 268 young women, the median duration of symptoms was 36 months (IQR 24-84), top five most commonly reported symptoms were: sensation of a mass or bulge in the vagina (n = 225, 84%), lower abdominal pain (n = 70, 26.1%), lower back pain (n = 50, 18.7%), urinary incontinence (n = 35, 13.1%), and stool incontinence (n = 33, 12.3%). POP was most frequently in the posterior compartment (n = 59, 22%), combined anterior and apical compartments (n = 54, 20.2%), anterior compartment (n = 53, 19.8%), and found less frequently involving all three compartments (n = 21, 7.8%). Concurrent pelvic floor disorders were: obstetric anal sphincter injuries (n = 21, 7.8%) and stress urinary incontinence (n = 8, 3.0%) as shown in Table 2.

**Table 2:** Clinical presentation of POP in young women (n = 268) *Vaginal cyst (n=4), rectal prolapse (n=1), paraurethral gland abscess (n=1), labial lipoma (n=1), anterior abdominal wall incision hernia (n=1), ovarian cyst (n=1) POP: Pelvic organ prolapse; IQR: Interquartile range

Description of clinical presentation	Frequency (n = 268)	Percentage (%)
Duration of symptoms (months), median (IQR)	36 (24-84)	
Symptoms		
Mass prolapsing from the vagina	225	84
Lower abdominal pain	70	26.1
Lower back pain	50	18.7
Urinary incontinence	35	13.1
Stool incontinence	33	12.3
Painful sexual intercourse	28	10.5
Wide vaginal opening	17	6.3
Abnormal vaginal discharge	14	5.2
Vaginal bleeding	8	3
Prolapsed compartment(s)		
Posterior	59	22
Anterior and apical	54	20.2
Anterior	53	19.8
Apical	38	14.2
Anterior and posterior	28	10.5
Posterior and apical	15	5.6
All 3 compartments	21	7.8
Concurrent pelvic floor disorders/other gynecologic condition		
None	230	85.8
Stress urinary incontinence	8	3
Obstetric anal sphincter injuries	21	7.8
Other gynecologic/surgical conditions*	9	3.3

**Figure 3:**
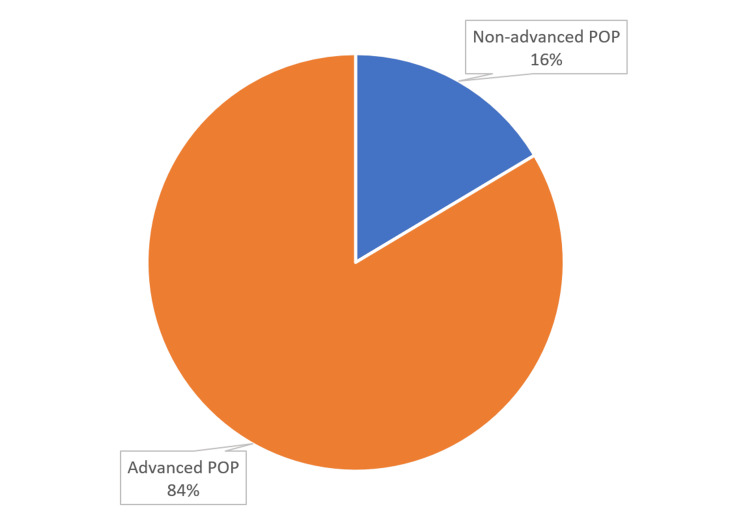
Prevalence of advanced (stage III & stage IV) POP among young women (n = 268) The prevalence of advanced POP was calculated as a percentage by dividing the number young women with stage III or IV POP (n = 224) by the sample size (N = 268). The figure was created in Microsoft Word on Windows 11. POP: Pelvic organ prolapse

Of the 268 young women, 224 had advanced (stage III or IV) POP, giving a prevalence of 84% (95% CI 78.63-87.57) as shown in Figure 3.

At bivariable analysis for socio-demographic, medical, obstetric and gynecologic factors, the factors found to be significantly associated with advanced POP were: residence, level of education, marital status, occupation, tobacco or cigarette smoking, parity, and prior POP as shown in Table 3.

**Table 3:** Bivariable analysis for socio-demographic, medical, obstetric, and gynecologic factors (n = 268) p-values obtained from modified Poisson regression analysis with robust standard errors. *p < 0.05; # Parity was analyzed as a continuous variable POP: Pelvic organ prolapse; PR: Prevalence ratio; CI: Confidence interval

Variable	Total (n = 268)	Advanced POP (n = 224)	Non-advanced POP (n = 44)	Unadjusted PR (95% CI)	p-value
Age categories					
< 40	126 (47.0)	105 (46.9)	21 (47.7)	reference	
≥ 40	142 (53.0)	119 (53.1)	23 (52.3)	1.01 (0.90-1.12)	0.918
Residence					
Urban	18 (6.7)	10 (4.5)	8 (18.2)	reference	
Rural	250 (93.3)	214 (95.5)	36 (81.8)	1.54 (1.02-2.34)	0.042*
Highest level of education					
No formal education	46 (17.2)	44 (19.6)	2 (4.6)	reference	
Primary	116 (61.9)	137 (61.2)	29 (65.9)	0.86 (0.79-0.95)	0.002*
Post primary	56 (20.9)	43 (19.2)	13 (29.6)	0.80 (0.69-0.94)	0.006*
Marital status					
Widowed	11 (4.1)	11 (4.9)	0 (0.0)	reference	
Single, divorced or separated	59 (22.0)	45 (20.1)	14 (31.8)	0.76 (0.66-0.88)	<0.001*
Married	198 (73.9)	168 (75.0)	30 (68.2)	0.85 (0.80-0.90)	< 0.001*
Occupation					
Others (small businesses, salaried jobs, etc.)	59 (22.0)	41 (18.3)	18 (40.9)	reference	
Peasant farmer	209 (78.0)	183 (81.7)	26 (59.1)	1.26 (1.06 – 1.50)	0.010*
Work involves lifting heavy loads (n = 264)					
No	58 (22.0)	45 (20.5)	13 (29.6)	reference	
Yes	206 (78.0)	175 (79.6)	31 (70.5)	1.09 (0.94- 1.27)	0.236
Hours of physical work					
0-3	40 (14.9)	33 (14.7)	7 (15.9)	reference	
4-6	168 (62.7)	150 (67.0)	18 (40.9)	1.08 (0.93-1.26)	0.309
7 and more	60 (22.4)	41 (18.3)	19 (43.2)	0.83 (0.66-1.04)	0.099
Tobacco or cigarette smoking					
Yes	2 (0.8)	2 (0.9)	0 (0.0)	reference	
No	265 (99.3)	222 (99.1)	43 (100.0)	0.84 (0.79-0.88)	< 0.001*
Parity mean (SD)^#^	5.6 (2.6)	5.8 (2.6)	4.6 (2.2)	1.03 (1.01-1.05)	0.004*
Vaginal delivery at home					
No	121 (45.3)	99 (44.4)	22 (50.0)	reference	
Yes	146 (54.7)	124 (55.6)	22 (50.0)	1.04 (0.93-1.16)	0.5
Prior POP surgery					
No	257 (95.9)	213 (95.1)	44 (100.0)	reference	
Yes	11 (4.1)	11 (4.9)	0 (0.0)	1.21 (1.14-1.28)	<0.001*
Menopause					
Yes	28 (10.6)	25 (11.3)	3 (7.0)	reference	
No	236 (89.4)	196 (88.7)	40 (93.0)	0.93 (0.81-1.07)	0.314
Duration of POP symptoms					
Less than 2 years	63 (36.8)	57 (38.8)	6 (25.0)	reference	
2 to 5 years	51 (29.8)	42 (28.6)	9 (37.5)	0.91 (0.78-1.06)	0.221
More than 5 years	57 (33.3)	48 (32.7)	9 (37.5)	0.93 (0.81-1.07)	0.31
History of hypertension					
No	225 (84.0)	189 (84.4)	36 (81.8)	reference	
Yes	43 (16.0)	35 (15.6)	8 (18.2)	0.97 (0.83-1.13)	0.689
History of diabetes mellitus					
No	262 (97.8)	221 (98.7)	41 (93.2)	reference	
Yes	6 (2.2)	3 (1.3)	3 (6.8)	0.59 (0.27-1.32)	0.202
BMI					
Normal weight (18.5-24.9)	36 (16.3)	32 (16.8)	4 (12.9)	reference	
Underweight (< 18.5)	115 (52.0)	99 (52.1)	16 (51.6)	0.97 (0.84-1.11)	0.647
Overweight & obese (≥ 25)	70 (31.7)	59 (31.1)	11 (35.5)	0.95 (0.81-1.11)	0.498
History of chronic cough (lasting 6 months or more)					
No	241 (92.3)	204 (93.6)	37 (86.1)	reference	
Yes	20 (7.7)	14 (6.4)	6 (14.0)	0.83 (0.62-1.12)	0.203

At multivariable analysis, history of prior POP surgery (adjusted prevalence ratio (aPR) 1.19 CI: 1.08- 1.31, p < 0.001) was associated with increased likelihood for advanced POP while having attained primary education (aPR 0.90 CI: 0.82-0.98, p = 0.015) and being married (aPR 0.91 CI: 0.85-0.98, p = 0.009) were protective against advanced POP (Table 4).

**Table 4:** Multivariable analysis for factors associated with advanced POP among young women (n = 268) p-values were obtained from modified Poisson regression analysis with robust standard errors. *p < 0.05; # Parity was analyzed as a continuous variable POP: Pelvic organ prolapse; cPR: Crude (unadjusted) prevalence ratio; aPR: Adjusted prevalence ratio; CI: Confidence interval

Variable	Bivariable analysis		Multivariable analysis	
	cPR (95% CI)	p-value	aPR (95% CI)	p-value
Parity^#^	1.03 (1.01-1.05)	0.004*	1.01 (0.99-1.03)	0.333
Prior POP surgery				
No	reference			
Yes	1.21 (1.14-1.28)	<0.001*	1.19 (1.08-1.31)	< 0.001*
Residence				
Urban	reference			
Rural	1.54 (1.02-2.34)	0.042*	1.41 (0.92-2.14)	0.114
Highest level of education				
No formal education	reference			
Primary	0.86 (0.79-0.95)	0.002*	0.90 (0.82-0.98)	0.015*
Post primary	0.80 (0.69-0.94)	0.006*	0.95 (0.80-1.14)	0.591
Marital status				
Widowed	reference			
Married	0.85 (0.80-0.90)	< 0.001*	0.91 (0.85-0.98)	0.009*
Single, divorced, or separated	0.76 (0.66-0.88)	<0.001*	0.89 (0.75-1.04)	0.152
Occupation				
Small businesses, salaried jobs, etc.	reference			
Peasant farmer	1.26 (1.06-1.50)	0.010*	1.10 (0.89-1.35)	0.369
Hours of physical work				
0 – 3	reference			
4 – 6	1.08 (0.93-1.26)	0.309	1.02 (0.88-1.17)	0.831
7 and more	0.83 (0.66-1.04)	0.099	0.89 (0.71-1.12)	0.320
Tobacco or cigarette smoking				
Yes	reference			
No	0.84 (0.79-0.88)	< 0.001*	0.78 (0.55-1.11)	0.169

## Discussion

Proportion of young women among POP surgical patients

This study found that nearly half (49%, 268/547) of women operated for POP were young women aged 18-49 years. A similar finding was reported in a retrospective records review study done at Jimma University Medical Centre, south-west Ethiopia, whereby 53.3% of women operated for stage III or IV POP were found to be within reproductive age (≤ 49 years) [5]. The occurrence of advanced POP in young women in low-resource settings in sub-Saharan Africa is probably driven by too early, too frequent and too many childbirths, that are most times unattended by skilled maternity care providers [5,6], early return to strenuous physical activity after childbirth, a high prevalence of maternal anemia, and malnutrition [11]. This is compounded by low level of education and relegation to a manual labor-intensive lifestyle in rural areas as peasant farmers [7,15]. High parity and inadequate obstetric care have been shown to cause pelvic connective tissue, muscle, and nerve injury, thereby weakening vaginal supports [1]. Occupations such as peasant farming and the rural lifestyle that involves lifting heavy weights of harvests, digging and household activities, increase intra-abdominal pressure straining the pelvic floor thereby leading to POP [5,12,16].

Duration of POP symptoms

Like previous studies from sub-Saharan Africa, a long duration of symptoms reflecting delay in seeking care for POP, was found in the current study [5,9]. The median duration of symptoms was 36 months (IQR 24-84). The delayed presentation has been attributed by previous researchers to stigma, socialization to normalize female genital tract alterations following delivery, limited awareness about POP, limited access to urogynecologic care, and depressive symptoms associated with pelvic floor disorders [6,9].

The most commonly reported POP symptoms in this study were: sensation of a mass or bulge in the vagina, lower abdominal pain, lower back pain, urinary incontinence, and stool incontinence. Sensation of a mass or bulge in the vagina is a commonly reported symptom of advanced POP in literature [14,15]. It is the specific symptom for advanced POP where the most dependent part of the prolapsed organ is below the hymenal remnants [15,17]. Contrary to this, an earlier study from Kampala, Uganda, found limited reporting of symptoms specific for POP probably because majority of the participants had less severe POP (stage II) [9]. Reporting of symptoms that are non-specific to POP can be attributed to: more bothersome concurrent pelvic floor disorders such as urinary or stool incontinence and probably avoidance of POP genital symptoms considered to be embarrassing.

Compartments frequently affected by POP in young women

Posterior and anterior combined with apical compartment, were more frequently affected by POP. Anterior and apical compartments are frequently and often times jointly prolapsed [1,5]. This is probably the result of gravity and downward drag of the anterior and apical compartment occupant pelvic viscera after they have lost anatomic support while the posterior vaginal wall remains deviated dorsally, resting on and attached to the pelvic diaphragm. In the current study, obstetric anal sphincter injuries that disrupt posterior vaginal wall support were prevalent and may in part explain the frequent posterior compartment prolapses found.

Concurrent pelvic floor disorders with POP among young women

Obstetric anal sphincter injuries and stress urinary incontinence occurred concurrently with advanced POP in some young women. Stress urinary incontinence was reported in three of 92 (3.3%) women with advanced POP in a study from south-west Ethiopia [5]. The finding of concurrent pelvic floor disorders emphasizes the shared risk factors and pathogenesis of these disorders, arising from vaginal birth incited endopelvic fascia, muscle and neurovascular injury.

Prevalence of advanced POP among young women

The prevalence of advanced POP among young women operated for POP in urogynecologic camps in Uganda was 84% (224/268, CI: 78.63-87.57).

A community-based survey in south-west Ethiopia reported a low overall prevalence of symptomatic POP (6.6%, 28/422) but the proportion of young women symptomatic for POP who were found to have anatomical POP upon pelvic examination was slightly higher than our finding (89.29%, 25/28) [14]. That study did not differentiate between the stages of symptomatic POP reported upon it could have included symptomatic stage II prolapse in computing the proportion of symptomatic POP. Our study builds onto previous studies by reporting on the stages or severity of POP among young Ugandan women using the internationally recognized POP-Q staging system [14].

The high prevalence of advanced POP among young women in our study is explained by the highly selected study population of women undergoing POP surgery, high mean parity of 5.6 (SD 2.6), majority of study participants were peasant farmers (78.0%), and reported doing work that involved lifting heavy weights (78.0%) such as crop harvests, firewood, and water which are common in rural areas in low income sub-Saharan countries like Uganda [7,12,16].

Factors associated with advanced POP among young women

We found that history of prior POP surgery increased the likelihood for advanced POP (p < 0.001) while having attained primary education (p = 0.015) and being married were protective (p = 0.009) against advanced POP.

Explanations have been put forward for the increased likelihood for POP after prior genital prolapse surgery by other researchers. Firstly, recurrence of prolapse in the previously operated pelvic compartment due to surgical failure (low efficacy or faulty surgical repair technique), inherent native tissue weakness, surgical complications such as surgical site infection or hematoma formation, omission of fixation of the vaginal cuff following transvaginal hysterectomy [1,18]. Secondly, recurrence of prolapse in a new compartment due to alteration of the vaginal axis after the initial POP surgery leading to transfer of strain to the newly affected compartment, and early return to domestic chores particularly among young African women from rural settings [11,13,18]. Previous studies have quoted recurrence POP to be 30% to 50% following vaginal native tissue repairs [1,2].

In general terms earlier studies collaborate the finding that education lowers the risk for POP [19,20]. However, this has been reported mainly for intermediate level and higher education [11]. This is probably through improved lifestyle, nutrition, and fewer childbirths. In the current study primary and post primary education were both protective against POP compared to no education at bivariable analysis but post-primary education became non-significant at multivariable analysis. This perhaps indicates that in this setting the initial more critical role is played by primary education and we hypothesize that this could be through delaying age at first childbirth. The contribution of other factors probably overrides the protective effect of education after primary level in this setting. The only other study accessible to us that has demonstrated a link between basic education and POP is from Bangladesh where researchers found that women whose husbands had five or more years of formal education were 63% less likely to have POP compared to those whose husbands had no education [21].

In the current study, women who identified as being married were 9% less likely to have advanced POP compared to widowed women. Women in union probably receive support from their partners reducing their exposure to strenuous physical activities in the homestead compared to their widowed counterparts. Additionally, married women in low-resource settings probably receive financial support from their partners and seek treatment early hence being protected from severe POP. Though we did not find a study collaborating this association from literature, an earlier study found that women without spouses were more likely to delay in seeking care for pelvic floor disorders [10].

Strength of the study

This is by far one of the few studies in sub-Saharan to describe the clinical presentation of, prevalence of, and profiles of young women of reproductive age with advanced POP with a substantial sample size, using multicenter data extracted from a surgical database. The findings can therefore be inferred to similar settings.

Limitations of the study

The study being a retrospective review of records only investigated a few risk factors for POP, and had missing data. Therefore, the study does not provide a complete view of the all the relevant factors that are associated with POP in young women. Self-reported data in this analysis could have been affected by recall bias however efforts were undertaken to collaborated the study participants’ data with their clinical or treatment records. Results should be interpreted with caution due to possibility of selection bias as the study population was restricted to only young women managed for POP surgically during surgical camps at specific MUST supported urogynecology centers in Uganda.

## Conclusions

We conclude that the prevalence of advanced stage POP among young women (18-49 years) undergoing POP surgery in urogynecologic camps in Uganda is high. The common presenting symptom is a vaginal bulge or mass, and the commonly encountered concurrent pelvic floor disorders are stress urinary incontinence and obstetric anal sphincter injuries. Prior POP surgery increases the likelihood for advanced POP while education and being married are protective. Given the high prevalence of advanced POP found by the current study among young women, the burden of POP, health expenditure on POP care, and repeat surgical operations for POP are likely to increase as the population ages. We, therefore, recommend intensifying preventive strategies against POP in young women such as scaling up family planning uptake, skilled obstetric care, and postpartum pelvic floor muscle rehabilitation. Feasibility studies should be undertaken to assess adoption of vaginal pessaries for management of POP in young women in Uganda since they have been shown to be an effective treatment elsewhere and they do not carry the risk of repeat surgery. We advocate for increasing access to universal education in underserved regions of Uganda and sub-Saharan Africa.

## References

[1] Weintraub AY, Glinter H, Marcus-Braun N (2020). Narrative review of the epidemiology, diagnosis and pathophysiology of pelvic organ prolapse. Int Braz J Urol.

[2] Lallemant M, Clermont-Hama Y, Giraudet G, Rubod C, Delplanque S, Kerbage Y, Cosson M (2022). Long-term outcomes after pelvic organ prolapse repair in young women. J Clin Med.

[3] (2019). Pelvic organ prolapse: ACOG Practice Bulletin, Number 214. Obstet Gynecol.

[4] Wang B, Chen Y, Zhu X (2022). Global burden and trends of pelvic organ prolapse associated with aging women: An observational trend study from 1990 to 2019. Front Public Health.

[5] Sori DA, Bretones S, Mellier G, de Rochambeau B (2022). Prevalence and surgical outcomes of stage 3 and 4 pelvic organs prolapse in Jimma university medical center, south west Ethiopia. BMC Womens Health.

[6] Scherf C, Morison L, Fiander A, Ekpo G, Walraven G (2002). Epidemiology of pelvic organ prolapse in rural Gambia, West Africa. BJOG.

[7] Merga A, Bidira K, Geda A, Nigatu D, Bayana E (2023). Pelvic organ prolapse and its associated factors among women: a facility based cross-sectional study. Inquiry.

[8] Kayondo M, Kajabwangu R, Kaye D (2026). Magnitude, presentation, and management of pelvic organ prolapse at a tertiary referral hospital in southwestern Uganda: retrospective medical records review (2014-2018). Cureus.

[9] Vemulapalli R, Ngobi MD, Kiweewa FM (2025). Pelvic floor disorder prevalence and risk factors in a cohort of parous Ugandan women. Int J Gynaecol Obstet.

[10] Adefris M, Abebe SM, Terefe K (2017). Reasons for delay in decision making and reaching health facility among obstetric fistula and pelvic organ prolapse patients in Gondar University hospital, Northwest Ethiopia. BMC Womens Health.

[11] Vergeldt TF, Weemhoff M, IntHout J, Kluivers KB (2015). Risk factors for pelvic organ prolapse and its recurrence: a systematic review. Int Urogynecol J.

[12] Tugume R, Lugobe HM, Kato PK, Kajabwangu R, Kanyesigye H, Masembe S, Kayondo M (2022). Pelvic organ prolapse and its associated factors among women attending the gynecology outpatient clinic at a tertiary hospital in southwestern Uganda. Int J Womens Health.

[13] Jones KA, Moalli PA (2010). Pathophysiology of pelvic organ prolapse. Female Pelvic Med Reconstr Surg.

[14] Badacho AS, Lelu MA, Gelan Z, Woltamo DD (2022). Uterine prolapse and associated factors among reproductive-age women in south-west Ethiopia: a community-based cross-sectional study. PLoS One.

[15] Siyoum M, Nardos R, Teklesilasie W, Astatkie A (2024). Prevalence and risk factors of pelvic organ prolapse among women in Sidama region, Ethiopia: a community-based survey. Womens Health (Lond).

[16] Masenga GG, Shayo BC, Rasch V (2018). Prevalence and risk factors for pelvic organ prolapse in Kilimanjaro, Tanzania: a population based study in Tanzanian rural community. PLoS One.

[17] Kayembe AT, Ilunga BM, Muakuya JM, Muela AM, Tozin RR (2024). Pelvic organ prolapse: a cross-sectional study during mass campaign in two hospitals in the city of Kananga in the Democratic Republic of Congo. Pan Afr Med J.

[18] Kayondo M, Geissbüehler V, Migisha R (2022). Risk factors for recurrence of pelvic organ prolapse after vaginal surgery among Ugandan women: a prospective cohort study. Int Urogynecol J.

[19] Progetto Menopausa Italia Study Group (2000). Risk factors for genital prolapse in non-hysterectomized women around menopause. Results from a large cross-sectional study in menopausal clinics in Italy. Eur J Obstet Gynecol Reprod Biol.

[20] Nygaard I, Bradley C, Brandt D (2004). Pelvic organ prolapse in older women: prevalence and risk factors. Obstet Gynecol.

[21] Akter F, Gartoulla P, Oldroyd J, Islam RM (2016). Prevalence of, and risk factors for, symptomatic pelvic organ prolapse in rural Bangladesh: a cross-sectional survey study. Int Urogynecol J.

